# Phylogeography of amphi-boreal fish: tracing the history of the Pacific herring *Clupea pallasii* in North-East European seas

**DOI:** 10.1186/1471-2148-13-67

**Published:** 2013-03-19

**Authors:** Hanna M Laakkonen, Dmitry L Lajus, Petr Strelkov, Risto Väinölä

**Affiliations:** 1 Finnish Museum of Natural History, University of Helsinki, POB 17, FI-00014 Helsinki, Finland; 2 Department of Ichthyology and Hydrobiology, St Petersburg State University, 16 Line, 29, Vasilevsky Island, St Petersburg 199178, Russia

**Keywords:** Phylogeography, Amphi-boreal fauna, White Sea, Trans-Arctic colonization, mtDNA, Time-dependent rates

## Abstract

**Background:**

The relationships between North Atlantic and North Pacific faunas through times have been controlled by the variation of hydrographic circumstances in the intervening Arctic Ocean and Bering Strait. We address the history of trans-Arctic connections in a clade of amphi-boreal pelagic fishes using genealogical information from mitochondrial DNA sequence data. The Pacific and Atlantic herrings (*Clupea pallasii* and *C. harengus*) have basically vicarious distributions in the two oceans since pre-Pleistocene times. However, remote populations of *C. pallasii* are also present in the border waters of the North-East Atlantic in Europe. These populations show considerable regional and life history differentiation and have been recognized in subspecies classification. The chronology of the inter-oceanic invasions and genetic basis of the phenotypic structuring however remain unclear.

**Results:**

The Atlantic and Pacific herrings both feature high mtDNA diversities (large long-term population sizes) in their native basins, but an ocean-wide homogeneity of *C. harengus* is contrasted by deep east-west Pacific subdivision within Pacific *C. pallasii*. The outpost populations of *C. pallasii* in NE Europe are identified as members of the western Pacific *C. pallasii* clade, with some retained inter-oceanic haplotype sharing*.* They have lost diversity in colonization bottlenecks, but have also thereafter accumulated abundant new variation. The data delineate three phylogeographic groups within the European *C. pallasii*: herring from the inner White Sea; herring from the Mezen and Chesha Bays; and a strongly bottlenecked peripheral population in Balsfjord of the Norwegian Sea.

**Conclusions:**

The NE European outposts of *C. pallasii* are judged to be early post-glacial colonists from the NW Pacific. A strong regional substructure has evolved since that time, in contrast to the apparent broad-scale uniformity maintained by herrings in their native basins. The structure only partly matches the previous biological concepts based on seasonal breeding stocks or geographical subspecies designations. The trans-Arctic herring phylogeography is notably similar to those of the amphi-boreal mollusk taxa *Macoma* and *Mytilus,* suggesting similar histories of inter-oceanic connections. We also considered the time dependency of molecular rates, critical for interpreting timing of relatively recent biogeographical events, by comparing the estimates from coding and non-coding mitochondrial regions of presumably different mutation dynamics.

## Background

The boreal faunas of the North Atlantic and North Pacific oceans comprise many instances of closely related, vicariously distributed species pairs, reflecting a history of shared ancestry followed by inter-oceanic isolation through the Pleistocene and Holocene epochs. In most cases, this vicariance is thought to trace back to the Great Trans-Arctic Interchange approximately 3.5 Mya that followed the Pliocene opening of the Bering Strait, until which most of the lineages were restricted to a single ocean basin, and after which the Pleistocene conditions again restricted the dispersal (e.g. [1,2]). Yet even since that time species could in principle have had several opportunities to disperse through the Arctic. The patterns of biotic exchange have been controlled by the history of climatic and hydrographical circumstances, but also by the thermal tolerance and dispersal characteristics of the taxa. Indeed, phylogeographical studies of amphi-boreal taxa have so far demonstrated a variety of inter-oceanic systematic affinities and more complex isolation/dispersal histories such as repeated trans-Arctic invasions, both in fishes (e.g. [3,4]) and invertebrates [5,6].

Among the most prominent pairs of amphi-boreal vicariant taxa are the Pacific and Atlantic herrings, *Clupea pallasii* Valenciennes, 1847 and *Clupea harengus* Linnaeus, 1758. They are pelagic planktivores occurring in massive schools and occupying both coasts of their respective oceans, from the temperate up to the subarctic zone. The inter-oceanic vicariance caused by the Arctic dispersal barrier is however not complete, but is broken by the presence of remote populations of *C. pallasii* in border waters of the NE Atlantic in Europe, particularly in the White and the south-eastern Barents seas. Also the Atlantic *C. harengus* penetrates these seas from the west, although does not spawn there [7,8]. Moreover, isolated occurrences of *C. pallasii* even further west in some Norwegian Sea fjords are known [9].

The European populations of *C. pallasii* demonstrate remarkable heterogeneity of their life histories (e.g. [10]). In the White Sea Gulf of Kandalaksha, a fast-growing summer-spawning form similar to typical Pacific herring is distinguished from a more abundant slow-growing form, which uniquely breeds under the ice in the spring. There are also a number of other herring stocks in other parts of the White Sea and in the south-eastern Barents Sea, which differ in their growth rates and spawning seasons. The origins and status of the seasonal vs. geographical breeding stocks have been debated for decades, but genetic data so far have yielded contradicting results on these issues [11,12]. At a broader regional scale in NE Europe, a subspecies-level division has generally been recognized, into the White Sea herring *C. pallasii marisalbi* Berg, 1923, and the Chesha–Pechora herring *C. pallasii suworowi* Rabinerson, 1927 in the south-eastern Barents Sea [7].

The timing and the geography of the origin of the European populations remain unexplored in phylogeographic terms, whereas allozyme and initial mitochondrial data have confirmed their Pacific species identity [8,12,13]. Based on paleogeographical facts, they have been thought to represent relict populations of a wider geographic distribution that existed along the Eurasian Arctic coast during warmer post-glacial times < 10 ky ago (e.g. [14]). The European outposts of a Pacific boreal taxon provide a platform to consider the dynamics of trans-Arctic connections in the context of climatic history, and should help to understand the biological consequences of the current climatic warming for integrity of biological diversity in the boreal and arctic seas (cf. [15,16]).

Here we use the genealogical information in mitochondrial DNA sequence variation to assess the demographic histories and sub-structuring of the amphi-boreal species of herrings at various temporal and geographical scales, and particularly to trace the history and status of the NE European outpost populations of *C. pallasii*. Recent studies of *C. pallasii* within the Pacific have corroborated a pronounced intra-basin east–west subdivision, but raised discussion of interpreting the demography from mitochondrial control region data [17,18]. No comparable broad-scale sequence data exist for the North Atlantic *C. harengus,* whereas it has appeared genetically relatively homogeneous on oceanic and regional scales in other genetic markers (e.g. [19-21], but see [22]). As a background, we first assess the mtDNA diversity of the two species in their native basins from comparable datasets. Then focusing on the inter-oceanic dispersal that should account for the presence of *C. pallasii* in Europe, three main hypotheses of invasion times are considered, i.e. pre-glacial (e.g. during Eemian interglacial period) approximately 120 kya, early post-glacial from the opening of the Bering Strait to the Holocene Thermal Maximum (12–5 kya), or a still more recent arrival or continued genetic exchange. We further assess the structure of the “invading” *C. pallasii* among and within the NE European seas, and document striking regional differences that contrast with the homogeneity of the herring stocks in their native basins. The data have implications on concepts of the systematics, breeding stocks and comparative genetics of *C. pallasii* itself, but are also of more general importance in the (comparative) framework of the history of boreal marine taxa with similar trans-Arctic distributions.

The dating of phylogeographical events on molecular grounds is a topic of contention due to the apparent time dependency of substitution rates (e.g. [23-25]), an issue that was also raised in previous herring work [25]. We assess further the implications of the time dependency for the clupeid history and for inferences of trans-Arctic phylogeography more generally, by employing two sequence fragments of the mtDNA with potentially different mutation dynamics and biases (coding and non-coding).

## Methods

### Samples

**Figure 1:**
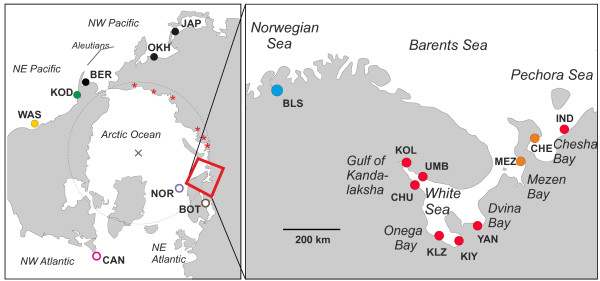
**Sampling localities for *Clupea pallasii *(dots) and *C. harengus *(circles).** For locality codes see Table 1. Color codes reflect genetic population groupings (cf. Figures 2, 3). Observations of historical herring occurrence along the Siberian coast in the warm period of 1930s-1940s [7] are indicated with stars.

Samples of *C. pallasii* were obtained between 1994 and 2010 from 16 locations, including five sites representative of the distribution in the Pacific-Bering Sea basins (American and Asian coasts) and eleven from NE Europe (Table 1, Figure 1). The latter included the four major bays of the White Sea, two locations from the eastern Barents Sea (= Pechora Sea) and an isolated fjord population from the Norwegian Sea (Balsfjord). The White Sea material covered several separate samples from the Gulf of Kandalaksha, including samples of the distinct spring-spawning herring. *C. harengus* samples represent extremes of its range: the Canadian coast, the Norwegian and the Baltic Seas. The samples were collected in accordance with the national legislation of the countries concerned. The samples were stored either in ethanol or frozen at −80°C.

**Table 1:** **Sample information for *Clupea pallasii *and *Clupea harengus***

**Taxon, area, location**	**Code**	***N***	**Date**	**Coordinates**
*Clupea pallasii*				
NW Pacific & Bering Sea				
Sea of Japan	JAP	24	2005	48.40°N, 141.94°E
Sea of Okhotsk, Taui Bay	OKH	37	5.2005	59.40°N, 150.12°E
			31.8.2007	59.18°N, 149.05°E
Bering Sea, Togiak Bay	BER	48		58°N, 160°W
NE Pacific				
Gulf of Alaska, Kodiak Bay	KOD	27		57°N, 154°W
Washington	WAS	28	2009	48°N, 125°W
Pacific, Total		164		
Pechora Sea				
Chesha Bay, Yarnei River	CHE	21	16.6.2010	67.78°N, 45.99°E
Indiga Bay, near Svyatoi Nos	IND	21	20.6.2010	67.87°N, 48.58°E
White Sea				
Mezen Bay, West Kanin	MEZ	24	16.6.2010	67.20 N, 43.49 E
Dvina Bay, Yandovaya Inlet	YAN	36+5	5.2001; 15.6.2010	64.66°N, 39.82°E
Onega Bay, Kolezhma Inlet	KLZ	21	11.5.2001	64.30°N, 35.91°E
Onega Bay, Kii Island	KIY	12	18.5.2001	64.00°N, 37.90°E
Gulf of Kandalaksha, Chupa Inlet*	CHP	10	19.4.2001	66.30°N, 33.47°E
Gulf of Kandalaksha, Chupa Inlet	CHU	23	30.8.1994	66.30°N, 33.47°E
Gulf of Kandalaksha, Kolvitsa*	KOL	38	30.4.2010	67.04°N, 32.55°E
Gulf of Kandalaksha, Umba	UMB	32	16.6.2010	66.65°N, 34.25°E
Norwegian Sea				
Balsfjord	BLS	39	28.4.2009	69.27°N, 19.28°E
European *C. pallasii*, Total		282		
*Clupea harengus*				
NW Atlantic, Canada	CAN	26	10.3.2010	47°N, 47°E
Norwegian Sea	NOR	26	2010	72°N, 16°E
Baltic Sea, Bothnian Sea	BOT	30	2009	62°N, 19°E
Total *C. harengus*		85		
Total, both species		528		

*Kandalaksha spring spawning herring.

### Molecular analysis

Total genomic DNA was extracted either from muscle or fin tissue or from single spawned fertilized eggs using a salt precipitation method [26] or a silica binding procedure in a plate format [27]. Two fragments of the mitochondrial genome were amplified and sequenced: the nearly complete *cytochrome b* gene (*cyt-b*, 1131 bp in final analysis), and the 5’-end of the *control region* (*CR*, 481 bp; details in Additional file 1). For both markers, sequence data were obtained from the same 528 fish altogether.

The data from the two gene regions were treated both separately and together as a concatenated data set. The diversity and genealogical relationships among mtDNA haplotypes were first illustrated with neighbor-joining trees, from pairwise distances between haplotypes estimated in PAUP* 4.0 [28] under the GTR+I+Γ model of nucleotide substitution (model selection and parameters, see Additional file 1). Mean pairwise distances within and between the two species were calculated using MEGA 5.01 [29]. For a subset of data, i.e. the mtDNA clade judged to have been involved in the trans-Arctic dispersal and subsequent connections, a 99% plausible parsimony network of haplotypes was constructed for the concatenated data set using the TCS 1.21 software [30].

Standard intrapopulation molecular diversity statistics were calculated using DnaSP v5 [31]. Genetic differences among geographic regions and among populations within regions were assessed using the *Φ*_ST_ statistics of the analysis of molecular variance (AMOVA) using the Arlequin software (version 3.5.1.2), for the concatenated data [32]. Inter-population relationships were illustrated by metric multidimensional scaling (MDS) from the pairwise *Φ*_ST_ distance matrix, using NTSYS-pc software [33]. Signals of past population expansions within population groups judged to represent historically coherent entities were illustrated in mismatch distributions separately for the two genes [34]. Changes in effective population size (*N*_e_) were further studied by coalescence simulations using Bayesian skyline plot analysis (BSL) with BEAST v1.5.3 software ([35]; details in Additional file 1).

**Figure 2:**
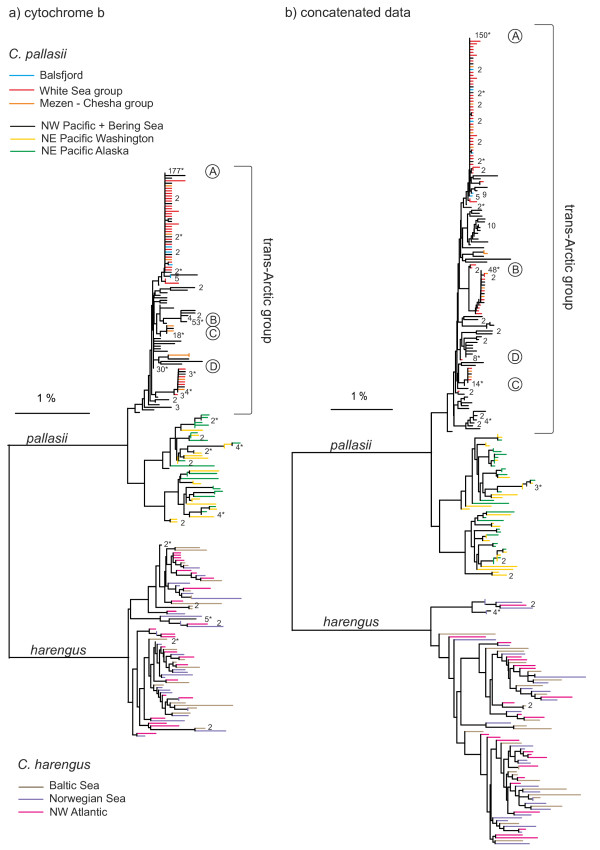
**Mitochondrial diversity in two herring species.** Neighbor-joining trees from GTR+I+Γ distances (**a**) from *cyt-b* data only (1131 bp) (**b**) from concatenated *cyt-b*+*CR* data (1617 bp). The numbers are observed frequencies of haplotypes found in multiple individuals; haplotypes shared between localities are indicated with asterisks. Color codes refer to population groups as in Figure 1, core haplotypes A-D as in Figure 3.

To estimate the initial trans-Arctic invasion time for *C. pallasii* in NE Europe, we used the Markov chain Monte Carlo coalescence model simulation implemented in the program IM (version updated in 12/17/2009) [36] on a subset of the *cyt-b* data representing the NW Pacific - Bering Sea region and the NE European populations, which formed a collective lineage exclusively shared by these regions (see Figure 2). The IM model assumes an ancestral population splitting at time *t* into two descendant populations of unequal size, subsequent divergence and exponential growth, and potentially asymmetric gene exchange. Based on sequence data of the two descendant populations the simulation generates posterior probability distributions for a number of demographic parameters: effective population sizes (scaled to gene mutation rate) following and preceding the divergence, the split proportion, reciprocal migration rates and split age. As there was differentiation among the European samples, data from genetically homogeneous subgroups of them were each separately contrasted with the samples from the Bering Sea and Asian Pacific.

We also used a rough direct count approach to estimate invasion age from the number of new mutations that were inferred to have arisen within the European populations since a putative founding bottleneck and population expansion, as seen in the haplotype network.

Conventionally, divergence rates used for molecular dating have been based on "deep" reference dates of pre-Pleistocene age, but in reality, on the more recent time scales dealt with here, the apparent time dependency of molecular rates becomes a major issue disturbing the linear time relationship; applying deep calibrations will provide overestimates of the ages of more recent events (e.g. [23,24]). Nevertheless, to facilitate description of the results, we will first use a tentative operational *cyt-b* rate of 1.5% My^-1^ (0.75% per lineage), in line with deep calibrations used for other fishes and with the basic trans-Arctic interchange/vicariance hypothesis (see Additional file 1).

## Results

### Intra- and interspecies sequence diversity

Extensive sequence variability was found in both gene regions, in both the Pacific and Atlantic herrings. The two species themselves were distinguished by an average 4.7% estimated sequence distance in *cyt-b* (GTR+I+Γ model; observed distance 3.6%) and estimated 16.9% distance in the *CR* sequence (observed 8.3%; Table 2). Within the Atlantic herring (*N* = 85), nearly all individuals had different haplotypes (*h* = 0.996/0.997/0.998 for *cyt-b*/*CR*/concatenated data) and the nucleotide diversities were *π* = 0.99%/2.85%/1.54% respectively. In the Pacific herring of the Pacific basin, similarly *h* = 0.96/0.97/0.98, *π* = 0.87%/1.95%/1.19% (*N* = 164). The genetic diversity estimates in the European outpost populations of the Pacific herring were considerably lower, *h* = 0.63/0.54/0.74 and π = 0.23%/0.35%/0.27% (pooled European samples, *N* = 282).

**Table 2:** Estimates of genetic diversity and coalescence times in two herring species and in selected population groups

		**Cytochrome b**			**Control region**			**Concatenated data**		
**Taxon / genetic group**	***N***	***h***	**π (%)**	**GTR distance (%)**	***h***	**π (%)**	**GTR distance (%)**	***h***	**π (%)**	**GTR distance (%)**
*C. harengus*										
All	85	0.996	0.99±0.13	1.07	0.997	2.85	3.63	0.998	1.54	1.78
*C. pallasii*										
Pacific	164	0.959	0.87±0.14	0.97	0.970	1.95	2.37	0.983	1.19	1.40
NE Pacific	55	0.989	0.92±0.14	1.03	0.981	2.17	2.64	0.997	1.29	1.52
NW Pacific	109	0.910	0.40±0.07	0.42	0.940	1.09	1.24	0.963	0.60	0.65
Europe	282	0.631	0.23±0.07	0.25	0.541	0.35	0.38	0.735	0.27	0.29
Mezen–Chesha group	45	0.811	0.41±0.11	0.44	0.748	0.54	0.58	0.834	0.45	0.48
White Sea group (incl Pechora Indiga)	198	0.565	0.19±0.06	0.20	0.442	0.29	0.31	0.650	0.22	0.23
Balsfjord	39	0.475	0.05±0.02	0.05	0.440	0.10	0.10	0.752	0.06	0.06
UPGMA basal distance										
*C. harengus* vs.Pacific *C. pallasii*			3.61	4.67		8.34	16.9			
TMRCA (My)				3.1			3.1			
*C. harengus*			1.43	1.57		3.82	5.04			
TMRCA (My)				1.0			0.9			
*C. pallasii*			1.35	1.54		2.86	3.59			
TMRCA (My)				1.0			0.7			

*h* - haplotype diversity, π - nucleotide diversity (= mean pairwise distance, observed; standard errors based on bootstrapping), mean pairwise GTR distance. UPGMA basal distances with corresponding TMRCA ages (Time to Most Recent Common Ancestor), based on the operational *u=*0.75 My^-1^ lineage^-1^ site^-1^ rate.

In the text below, usually either the *cyt-b* or concatenated datasets are described, whereas comparisons between different datasets are considered in the demographic analyses (mismatch distributions and Bayesian skyline plots), in Table 2, and in Additional file 2: Figure S1 and Additional file 3: Table S1. The information contents of the *cyt-b* and *CR* data sets alone were similar to each other, but the evolution of *cyt-b* is thought to be more regular and more amenable to model corrections. The *cyt-b* vs. *CR* comparisons will be taken up in the Discussion while considering the effects of the apparent time-dependence of molecular rates.

### Geographic structuring vs. homogeneity within ocean basins

In *C. pallasii* of the Pacific basin, the mtDNA variation was organized into three distinct clusters or lineages, in accord with previous *CR* data [17,18]. The primary division was between a North West Pacific (NWP) lineage that included the Russian Pacific and Bering Sea samples versus a NE Pacific (NEP) lineage, composed of North American samples south of the Alaska Peninsula. The NEP lineage was further subdivided into two clusters which were however geographically intermixed between the samples from Washington and Gulf of Alaska (WAS, ALA) (Figure 2). The two NEP clusters appeared reciprocally monophyletic in the *cyt-b* data and in the two-gene concatenated dataset, which was not evident from the *CR* data alone (Additional file 4: Figure S2). There was no significant differentiation between the two NEP lineage samples in terms of AMOVA apportionment of nucleotide diversity (*Φ*_ST_ = −0.002 for the concatenated dataset), neither among the four samples of the Asian coast and the Bering Sea that made up the NWP lineage (*Φ*_ST_ = 0.005; Table 3). The basal distance (coalescence) between the two main Pacific lineages, from simple UPGMA averaging, was 1.54% in *cyt-b* (GTR+I+Γ), i.e. 33% of the interspecies distance.

In the material from *C. harengus*, no geographical structuring was seen at the level of nucleotide diversity (*Φ*_ST_=-0.005 in AMOVA); haplotypes from the NW Atlantic, Norwegian Sea and Baltic Sea were mixed within the genealogy (Figure 2, Additional file 4: Figure S2). The basal *cyt-b* distance, 1.57%, was similar to that in the Pacific herring.

### Relationships between the NE Atlantic and North Pacific *Clupea pallasii*

**Figure 3:**
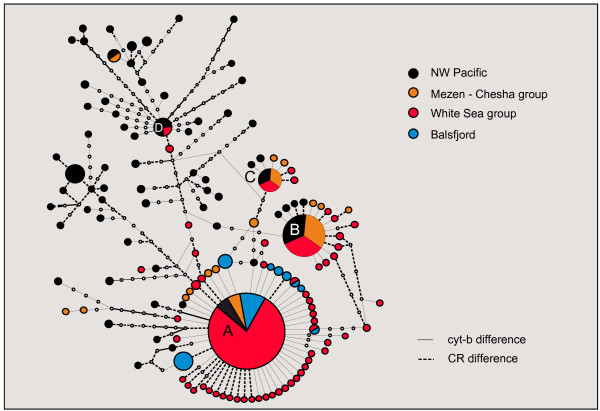
**Statistical (99%) parsimony network of haplotypes in the trans-Arctic *Clupea pallasii *mtDNA clade.** Concatenated *cyt-b*+*CR* data. Color codes refer to population groups as in Figure 1. Solid line refers to substitution in *cyt-b*, dashed line refers to substitution in *CR*.

The *C. pallasii* haplotypes from all the European samples clustered within the NWP lineage elsewhere distributed in the Bering Sea and Asian coasts (A lineage in [17]). This shared lineage will henceforth be referred to as the “trans-Arctic group” (Figure 2). The European samples did not comprise a separate monophyletic cluster within this group. They however did show distinctly lower intra-population and regional nucleotide and haplotype diversities than the Pacific relatives (Table 2). The European diversity mainly comprised of three or four dominant haplotypes (A-D) associated with sets of unique satellite haplotypes, one or two mutations away from the dominant ones (Figure 3). The same dominant haplotypes were also found in the North-West Pacific, where they similarly made nodes for local star-phylogenies in the network and were among the most abundant haplotypes.

### Diversity and differentiation within European *C. pallasii*

**Figure 4:**
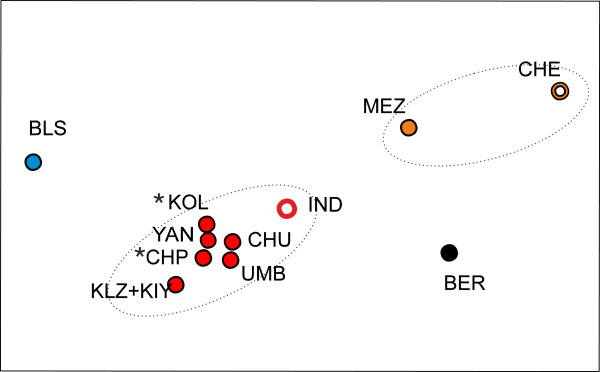
**MDS ordination of the NE European *Clupea pallasii *samples and one from the Bering Sea.** Ordination based on pairwise inter-population *Φ*_ST_ distances from the concatenated *cyt-b*+*CR* sequence data. For locality codes see Table 1. Kandalaksha spring spawning populations are indicated with asterisks. Color codes refer to population groups as in Figure 1.

We found strong geographical differentiation among the various *C. pallasii* samples of the North European seas (*Φ*_ST_ = 0.229, *P* = 0.00; Table 3). Using pairwise *Φ*_ST_ estimates for population clustering, the European samples may be divided into three relatively clear groups that show no significant internal heterogeneity (Figure 4). The grouping chiefly reflects the frequency variation of the four dominant haplotype clusters (illustrated in Figure 3): (1) Balsfjord group: the Norwegian Balsfjord (BLS) population, characterized by a single dominant haplotype A and its immediate satellites. (2) White Sea group: all the inner White Sea populations (Dvina Bay, Onega Bay and Gulf of Kandalaksha) along with the Indiga Bay sample (IND) from the Pechora Sea (*Φ*_ST_= 0.004, *P* = 0.28). Apart from the dominant haplotype clusters, five more distinct haplotype groups not closely related to them were recorded. (3) Mezen–Chesha group: the Mezen Bay sample (MEZ) along with the Pechora Sea sample from the Chesha Bay (CHE) (*Φ*_ST_ = 0.028, *P* = 0.17). This group overall has the most even representation of three core haplotypes A, B, C and their satellites, plus further three, rare haplotype groups distinct from the dominant ones. Notably, the two main population groups (2) and (3) are not geographically disjunctive but interlaced.

**Table 3:** **Estimates of the inter-population component of nucleotide diversity *Φ***_**ST **_**in geographically and genetically defined population groups of two herring species, from the concatenated *cyt-b *+ *CR *sequences**

**Taxon / genetic group**	***k***	***N***	***Φ***_**ST**_	***P *(*Φ***_**ST**_**=0)**
*C. pallasii*				
Pacific	5	164	0.356	0.00
NE Pacific	2	55	−0.002	0.40
NW Pacific	3	109	0.005	0.24
Europe	10	282	0.229	0.00
Mezen–Chesha group	2	45	0.024	0.17
White Sea group (incl Pechora Indiga)	8	198	0.004	0.28
*C. harengus*				
Atlantic	3	85	−0.005	0.66

*k –* number of geographical samples in the group, *N –* total number of individuals.

**Figure 5:**
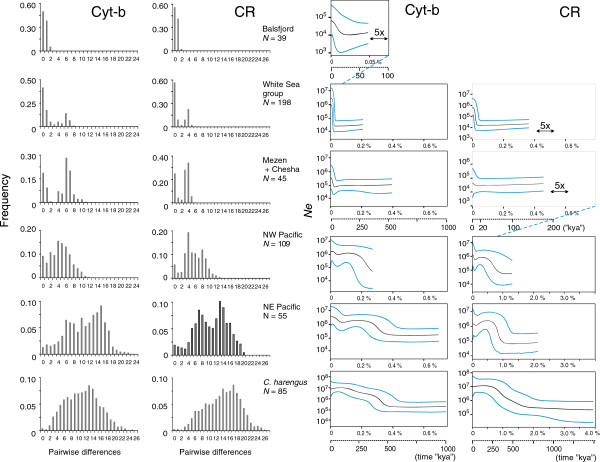
**Mismatch distributions and Bayesian skyline (BSL) plots reflect population size history.** Mismatch data in the two left panels, BSL plots in the two panels to the right. Results are presented for various putatively demographically coherent subsets of the data, for the *cyt-b* and *CR* data separately. Divergence and age estimates of inferred demographic events evaluated from these plots are presented in Additional file 3: Table S1. The BSL scales for *N*_e_ (y-axis) and operational timescales (x-axis) are based on 0.75% My^-1^ (*cyt-b*) and 2.7% My^-1^ (*CR*) per site mutation rates and a 4-year generation interval (see Additional file 1). Black lines are median estimates of *N*_e_, blue lines are the 95% HPD limits. End points of the lines represent the median estimate of the basal coalescence.

In terms of nucleotide diversity *π*, there was a progression among the three groups from a low 0.06% in (1) to 0.22% in (2) and to 0.45% in group (3) (concatenated data, Table 2; Figure 5).

### Signatures of demographic history

Mismatch distributions and BSL trajectories for selected subsets of the data, each judged to represent a coherent demographic history based on the geographical settings and homogeneity in AMOVA, further illustrate the patterns of nucleotide diversity (Figure 5, Additional file 3: Table S1). Both *C. harengus* and NE Pacific *C. pallasii* show broad dome-shaped mismatch distributions that can be interpreted as reflecting ancient demographic expansion events. For both, the main peaks are at approximately 13–15 *cyt-b* differences (0.57–0.67% change per lineage, uncorrected). The distributions are not simply unimodal (and thus not amenable to simple expansion model fitting), but secondary peaks are seen at 6–7 differences (ca. 0.30% change per lineage). The NW Pacific distribution in turn peaks at 4–6 differences. The distributions for the two main European groups are distinctly bimodal, with one mode at 6–8 similar to that in the NWP, and another one at zero, reflecting the dominant core haplotypes. Finally the Balsfjord distribution is L-shaped, with a zero peak reflecting the single dominant haplotype with satellites 1–2 mutational steps away.

The BSL plots suggest the first signal of coalescence for both *C. harengus* and NEP *C. pallasii* at 0.69–0.74% *cyt-b* per-site mutation units ago, and subsequent episodes of population growth at 0.30–0.40%, in agreement with the mismatch analysis (Figure 5, Additional file 3: Table S1). The youngest growth signal in the NE Pacific plot is at 0.05%, not apparent from the mismatch distribution. In the White Sea and Mezen–Chesha *C. pallasii*, stark signals of expansion are seen at 0.03% *cyt-b* units, corresponding to the most recent (zero) peak of the mismatch distribution.

### Trans-Arctic connections

The trans-Arctic invasion time for *C. pallasii* in NE Europe and subsequent migration rate were estimated using the IM model from the *cyt-b* data. Considering the genetic heterogeneity among the European populations, the three genetic clusters were each separately contrasted with the pooled sample from the NW Pacific. The highest posterior density estimates for the time-since-split were in the range *t* = 0.43–0.59 *cyt-b* per-gene mutation units, whether contrasting the NWP with the Mezen–Chesha group or with the White Sea group (Table 4 and Additional file 3: Table S2; 90% HPD intervals 0.25–1.77), which would correspond to 51–70 ky since the divergence with the conventional 0.75% per lineage per site rate. The founding Mezen–Chesha or White Sea population would have been ca. 1% of the ancestral population size, and for Balsfjord only 0.05% (Table 4). The estimates of post-split gene flow eastwards towards the White Sea (*m*_1_; ratio of immigration rate to mutation rate) were practically zero, whereas significant gene flow westwards to the NW Pacific was implied, amounting to *m*_2_ = 1–5, i.e. at a rate similar to the per-gene mutation rate (Table 4).

**Table 4:** **Estimates of ancestral population variability (*θ = N***_***e***_**μ), splitting parameter s, post-splitting migration rates (*m***_***1 ***_**= migration to NWP, *m***_***2 ***_**= migration to Europe) and parameter of time since divergence *(t) *obtained from coalescence-based IM model runs of the trans-Arctic clade**

**NW Pacific vs.**	***θ***_***A***_	**1*-s***	***m***_**1**_	***m***_**2**_	***t***	**(90% HPD)**
- White Sea group^a^	74	0.85%	2.98	0.003	0.44	(0.30–0.63)
- White Sea group^b^	54	1.85%	1.33	0.138	0.59	(0.56–1.49)
- Mezen–Chesha group	83	1.15%	4.99	0.003	0.43	(0.25–1.50)
- Balsfjord^a^	41	0.05%	0.65	0.003	*	-

The three genetically homogeneous European subgroups were individually compared with the pooled NW Pacific samples. More details in Additional file 3: Table S2.

^a^ Upper limits for prior distribution of *θ* set to 1000.

^b^ Upper limits for prior distribution of *θ* were set to *θ*_NWP_=1000, *θ*_Eur_=100 and *θ*_A_=100.

* The analysis did not converge.

In an alternative and informal approach, the time since invasion was evaluated directly from the number of inferred new mutations, which are illustrated in the haplotype network where two European core haplotypes are associated with several satellite haplotypes each (Figure 3). Assuming a sudden expansion at time *t* (star-coalescence), per-gene mutation rate *μ* and no drift or migration since then, we would expect *n* = *t*·*μ*·*N* new mutations in a sample of *N*. From the network, we count 34 putative mutations among 198 White Sea group individuals in the *cyt-b* sequence, which, given *μ* = 0.75·10^-8^·1131 bp, would be expected in *t* = 34/(*μ*·198) ≈ 20 ky (a minimum estimate). Similarly for the Mezen–Chesha group *t* ≈ 26 ky (=10/(*μ*·45)).

## Discussion

### Divergence and comparative intra-basin phylogeography of Pacific and Atlantic herrings

We estimated the inter-species sequence divergence between Atlantic and Pacific herring mtDNA lineages (*C. harengus* vs. *C. pallasii*) as 4.7% for the *cytochrome b* gene and 16.9% for the *CR* segment using appropriate substitution models, or 3.6% and 8.3% in terms of net distance, appropriate for assessing species divergence. These figures fit with the conventionally assumed vicariance history implied for taxa involved in the Pliocene Trans-Arctic Interchange [1,2], and with the suggested fish molecular rates. They are also compatible with the tentative 0.75% My^-1^ per lineage *cyt-b* rate used for the technical basis of discussion here (see Additional file 1).

Our data corroborate a fundamental geographical subdivision of *C. pallasii* within the Pacific basin, into an Asian–Beringian NWP lineage and a North American NEP lineage, found south of the Alaska Peninsula [17,18,37,38]. The NEP lineage is further subdivided into two clusters, but with no geographical structuring; it is controversial whether this reflects a cycle of refugial isolation and remixing [17] or just random mtDNA coalescence within a single panmictic, large and old population [18].

In contrast to the Pacific sister species, the Atlantic herring mtDNA data show no prominent signs of geographical subdivision even on a broad trans-oceanic scale. This is consistent with previous data on homogeneity in the bulk of other markers (e.g. [19-21]), whereas local differentiation at putatively selected loci have also been reported in recent genomic studies [22,39]. The broad-scale homogeneity can reflect effective long-term connectedness through the range, but it could also result from a history where the species was regionally extirpated from one side of the Atlantic temporarily and was then re-established by post-glacial trans-Atlantic invaders (cf. [19]). A history of single-coast Atlantic survival has been suggested for several boreal benthic invertebrates [40], and genetically corroborated for some [41]. The fish species capelin and cod in turn seem to have persisted through the LGM on both sides of the Atlantic [4,42].

Both herring species show remarkably high levels of sequence diversity, and those in Atlantic herring (*cyt-b π* = 0.90–1.03%) are much higher than in other widespread boreal Atlantic fishes considered to represent similar Atlantic–Arctic ancestries (e.g. capelin *cyt-b π* = 0.30–0.50%, [4]; Atlantic cod *CR π* = 0.17–0.63%, [43]). Yet disregarding the geography, the overall patterns in the two herring species*,* as reflected e.g. in the mismatch distributions, appear notably similar. The deep diversity itself implies large long-term population sizes, and peaked mismatch distributions imply an ancient expansion at the base of the genealogy (not recoverable by the BSL approach alone). The corresponding mtDNA TMRCA (illustrated as the start of the BSL graph) within each species is about 30% of the interspecies mtDNA divergence. These basal expansion signals could plausibly represent events during the early Middle Pleistocene climatic cycles ≤1 Mya (the time-dependency of rates should not bias estimates too much at this deep level, e.g. [24]). Traces of further growth episodes are also visible similarly in both species (Figure 5). A difference in the patterns is noted in a final signal of post-glacial growth only recorded in the *C. pallasii* NWP clade (cf. [18]) .

In assessing Pacific *C. pallasii* demography*,* Grant *et al*. [18] noted that recent bottlenecks (e.g. LGM) would wipe out evidence of older events in the BSL plots. However, in our data pre-LGM expansion signals are prominent in the NWP and NEP groups and in *C. harengus*, not only in mismatch distributions but also in the BSL plots (Figure 5). The end point of the BSL graph (the basal coalescence) may also represent an expansion, depending on the shape of the genealogy (cf. the mismatch distribution expansion peak). Nevertheless, it is notable that no signs of population bottlenecks over the Pleistocene glacial cycles are evident in the oceanic populations.

### Diversity and origin of the European *C. pallasii*

The European *C. pallasii* grouped tightly within the NWP lineage of the Pacific herring of the Bering Sea and the Asian Pacific coast (Figure 2), and the most common haplotypes were shared between the European and NWP stocks (Figure 3). The inter-oceanic connections have evidently been much younger than the intra-oceanic split of the NWP and NEP lineages discussed above.

#### A bottleneck history of European invaders

The European outposts show reduced mtDNA variation particularly in terms of haplotype diversity, with a strong dominance of 1–3 core haplotypes in each population (Table 2, Figure 3). Evidently only a small number of females effectively contributed to the colonization of Europe. A bottleneck signature is also seen in the IM results, suggesting founding population sizes 0.05–1% of the ancestral Pacific population (Table 4). The pattern of few dominant core haplotypes with closely associated satellites (new mutations) is a clear signature of demographic expansions following the invasion bottleneck [44], also reflected in the coalescence plots (Figure 5). More generally this bottleneck pattern corroborates the concept that the direction of colonization actually was from the Pacific towards Atlantic.

#### Estimating the trans-Arctic connections

Simulating the process of the split, divergence and accumulation of new mutations with the IM coalescence model, the age of the two-population split was estimated at approximately 0.5 *cyt-b* per-gene mutation units, corresponding to 50–70 ky under the operational 0.75% My^-1^ per site rate. The BSL analysis dated population expansions for European populations some 30 kya, whereas the direct count of inferred post-colonization mutations suggested dates between 20–26 kya, in accord with the mismatch estimates of 18–26 kya (Additional file 3: Table S1) from a similar reasoning.

While age estimates from the different approaches vary (18–70 kya, all based on the same *cyt-b* rate), they all inconveniently point to the Middle to Late Weichselian glacial times, and do not directly fit any of our *a-priori* dispersal time hypotheses. During this time the Bering Strait was effectively closed (e.g. [45]), and at any rate the paleoclimatic data indicate that conditions at these latitudes have been too cold for boreal species through Late Pleistocene interstadials [46]. The abundance of new mutations in itself directly rules out a hypothesis of recent, historical-time relationships (< 1 kya). At the same time, the time dependency of molecular rate (see below) implies that dates in this age frame will generally be gross overestimates rather than underestimates, thus the hypothesis of an earlier, Eemian interglacial ancestry (120 kya) can be confidently rejected. Considering the biological and paleoclimatic evidence, and concept of time-dependent rates, the invasion of *C. pallasii* to Northern Europe can then reasonably only be attributed to the Holocene since the opening of the strait (<12 kya), including the “Thermal Maximum” (HTM 5–8 kya; e.g., [47]).

The differences between the various divergence estimates from same data may reflect the differences in model assumptions concerning post-colonization migration and population size. Yet while IM suggested practically unidirectional eastward gene flow, the biological significance of the estimates is not entirely clear. The estimated gene flow rate was similar to the effect of mutations (*m*_*1*_ ~1–5; Table 4), which appears biologically unrealistic considering the assumed large population size of the recipient NWP–Bering Sea herring stock. At the same time, the presence of several unique non-core haplotypes in the European stocks could be seen as evidence of significant post-bottleneck immigration to the opposite direction. Evidently caution is needed when considering the IM migration estimates and other parameters given that they are based on coalescence simulation of a single locus.

Some support for continued trans-Arctic connections is however provided in records of herring populations found during the comparatively warm years of the 1930s-1940s at several Arctic Siberian sites (Figure 1: estuaries of the Ob, Enisei, Lena and Indigirka rivers; [7]). While the original invasion route for the European stocks itself remains uncertain, the source of those recent Siberian populations is of interest considering the direction and effects of potential long-term genetic exchange. Both the coastal currents [48] and the IM estimates support west-to-east gene flow. A biological hypothesis in line with this is that the European *C. pallasii* may be better adapted to Arctic conditions and thus more apt to disperse, since they descend from fish that successfully once crossed the Arctic; the Bering Sea fish in turn seem to make part of a widespread genetically uniform NW Pacific population including southern latitude stocks. Considering the potential effects of future climatic warming, such directionality would imply relatively minor genetic effects to the European stocks.

#### Systematics of *C. pallasii*

The *C. pallasii* mtDNA genealogy does not comply with the current subspecies division into a Pacific *C. p. pallasii* and two European taxa, *C. p. marisalbi* and *C. p. suworowi*. Rather, *C. p. pallasii* as currently defined is a paraphyletic unit, from which the putative European taxa arose; this is also evident in allozyme data [8]. The basic genetic division of *C. pallasii*, in both mitochondrial and nuclear data, is into a biologically comparatively homogenous American lineage and a more heterogeneous Eurasian lineage. The latter comprises both the Asian Pacific Bering Sea populations and the European outposts, representing all the three conventional subspecies. This Eurasian lineage is identifiable with the nominate *C. p. pallasii* Valenciennes, 1847 (described from Kamchatka) whereas a classification reflecting biological and historical relationships would attribute the American herrings another name; at a subspecies level that would be *C. p. mirabilis* Girard, 1854, originally described from San Francisco as *C. mirabilis* (see [49]). The distributional limit and biological differences of these taxa have been repeatedly documented at the Aleutian chain [17,37,38].

#### Substructure among NE European populations

The estimates of inter-oceanic relationships above were based on contrasting the NWP “ancestors” with a genetically homogeneous subgroup of the European samples, mainly from the inner White Sea basin (Figures 1 and 3; red dots). We however found strong regional heterogeneity among the various NE European samples, which roughly fall into three genetic groups, characterized by successively lower levels of intra-population diversity (Table 2; Figure 4). This complex genetic structuring of the European *C. pallasii* is unusual in view of the relative genetic homogeneity of both herring species in their native ranges.

The distinction between the two major, geographically overlapping or interdigitated groups in the inner White Sea vs. Pechora Sea (Mezen–Chesha) is puzzling, and it is unclear at what stage the subdivision arose and how it is maintained. The simple *Φ*_ST_ divergence estimates give no suggestion that the White Sea group would have descended from the Pechora population through serial colonization (e.g. the Pechora stock is not situated between White Sea and Bering Sea in the MDS ordination, Figure 4). The IM and direct count approaches both suggest similar invasion times for the two groups if treated separately (see above). It seems reasonable to assume that the colonization of Mezen–Chesha and White Sea regions was a result of the same invasion from the NW Pacific in the early Holocene, and the current complex substructure arose during further regional refugial phases associated with post-glacial climatic fluctuations. Genetic drift and limited migration must have been the primary factors in generating the structure, while selection that restricts gene exchange between locally adapted stocks might also have contributed to its maintenance.

The main genetic subdivision in the North European data largely accords with the previously established biological division into the “White Sea herring” *C. p. marisalbi* and the “Chesha-Pechora herring” *C. p. suworowi* of the SE Barents Sea. The latter has been thought to encompass the Mezen Bay population at the White Sea entrance (e.g. [7,50]), which is also supported by allozyme [12] and osteological data (D.L. Lajus, unpublished observations). The discrepancy arises with the Indiga sample (IND), geographically within the range of the Chesha–Pechora herring but genetically associated with the inner White Sea samples in our data (Figure 4). Fish in this sample were in spawning condition, and similar to other Chesha–Pechora herring in their characteristic growth rate, and probably represent true heterogeneity of the breeding stock in that area.

At a further level of biological structuring, the inner White Sea herring (*C. p. marisalbi*) are differentiated into several geographical and temporal spawning stocks, whose history and status have long been debated (see [10]). Significant chromosomal variations in Robertsonian polymorphisms between the major bays of the sea but not between sympatric seasonal breeding stocks suggested that geographical differences, probably reflecting local (post-glacial) adaptation, are more fundamental than those between seasonal stocks, which only would have arisen later [10,11]. In contrast, allozyme data have shown stable differentiation between sympatric seasonal stocks in the Gulf of Kandalaksha: the spring spawners were closer to the Chesha-Pechora ("*suworowi*") than to the sympatric summer-spawning White Sea herring [12]. Our White Sea mtDNA data covered samples from both the geographical and seasonal spawning ranges, but failed to demonstrate differences either between the main spawning regions in the inner White Sea (Kandalaksha, Onega and Dvina bays), or between the Kandalaksha spring- and summer-breeding cohorts – in contrast to the distinction of the three broader North European groups (Figure 4).

#### Balsfjord herring: outpost of an outpost

Balsfjord of the Norwegian Sea has a distinct breeding population of *C. pallasii* that returns to spawn at the same shallows of the fjord every year [51,52]. This is one of but two recognized *C. pallasii* populations on the Norwegian coast. The diversity of the *C. pallasii* mtDNA lineage in Balsfjord shows extreme reduction from the putative ancestral variation of the trans-Arctic group (Figure 3). The Balsfjord haplotypes seem to be derived from a single surviving haplotype lineage, suggesting a scenario where very few females effectively participated in the Balsfjord colonization and no essential further input was received from the east since that. The basal haplotype A is one of the major ones in the White Sea–Pechora Sea region, and indeed the most dominant one in the White Sea basin: a serial colonization from those stocks following the initial European bottlenecks therefore seems plausible. Yet the frequency of inferred post-bottleneck mutations in Balsfjord (5 of 39 ~ 13% in *cyt-b*) is similar to those in the White Sea–Pechora Sea region, and the colonization events thus probably still represent the same time frame, i.e. early post-glacial. The Balsfjord herring has also acquired some new variation through introgression (data to be presented elsewhere), but this should not affect the inferences above about its ancestry based on the original mtDNA lineage.

### Comparative trans-Arctic phylogeography

Two vicariant herring species of Atlantic and Pacific origin now meet secondarily in marginal seas of the North Atlantic, in NE Europe. Understanding the history of this contact is of broader importance, as it has several zoogeographical analogues, probably with similar histories. These occur e.g. among cods (Gadidae), which, as *Clupea,* are thought to have initially invaded the Pacific from the Atlantic in the Pliocene [53], but then, according to genetic data, returned back to the Atlantic on at least two occasions: The Pacific cod (*Gadus macrocephalus*) invaded the NW Atlantic and gave rise to the Greenland cod (*G. ogac*), while the Alaska pollock (*Theragra* (= *Gadus*) *chalcogramma*) invaded NE Atlantic, recorded there as “*T. finnmarchica*” [54,55]. Both these taxa now co-occur with the Atlantic cod in the northern Atlantic. A recent Atlantic re-invasion from the Pacific has also been suspected for the circumpolar capelin (*Mallotus villosus*), with a western Pacific–Arctic lineage present in the Labrador Sea [4].

**Figure 6:**
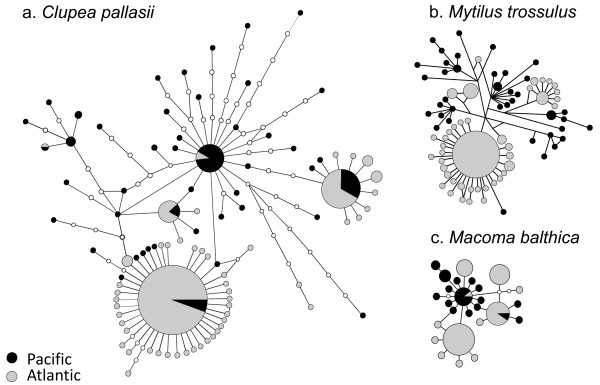
**Comparison of mitochondrial haplotype networks of trans-Arctic clades for three zoogeographically analogous amphi-boreal taxa.** (**a**) 99% statistical parsimony network of the trans-Arctic *Clupea pallasii* lineage (*cyt-b* data only). (**b**) Median-joining network of *Mytilus trossulus* (control region; redrawn from [6]).The branch lengths correspond to the number of substitutions but unobserved haplotypes are not marked here (**c**) 99% statistical parsimony network of *Macoma balthica* (COIII gene; [5]). Note the similar trans-Arctic distributions and signatures of expansion in the dominant Atlantic haplotypes.

In more detail, such repeated amphi-boreal connections have been studied with the molluscs *Macoma balthica* and *Mytilus* spp., which in turn are thought to represent primarily Pacific genera that first invaded the Atlantic in the Pliocene [5,6,56]. The re-invasions plausibly took place after the LGM either to the NE or NW Atlantic, where the newly invading lineages now coexist (and hybridize) with earlier established Atlantic taxa. In contrast to the herring of NW Pacific ancestry, the re-invading *Mytilus* and *Macoma* however have tight affinities to the NE Pacific coast. The genetic structures of the trans-Arctic *Macoma balthica* clade and *Mytilus trossulus* are strikingly similar to that of *C. pallasii* in terms of the mtDNA haplotype networks (Figure 6): the diversity of post-glacial Atlantic groups is concentrated in a few core haplotypes representing a fraction of that in the ancestral Pacific population, and accompanied by several rare satellite haplotypes derived following the founding bottleneck. The amount of post-colonization diversity (scaled by assumed relative gene mutation rate) is similar in all three taxa, reflecting early post-glacial invasion age. Unlike the herrings, the amphi-boreal bivalve sister taxa remain morphologically cryptic. It remains to be explored how general and important such repeated trans-Arctic contacts have been in shaping the general diversity in the amphi-boreal fauna.

### Non-linear rate and amending of time estimates

The effect of the apparent time-dependency of molecular rate to estimates of demographic events on phylogeographical time scales has been considered recently by several authors (e.g. [23-25]). As a general observation, molecular change on shorter time scales appears to occur at a faster rate than if averaged over phylogenetic scales. Conventional rate estimates are typically based on fossil or biogeographical “calibration” dates in a > 2 My time frame, and if directly applied to recent phylogeographic events will result in age estimates several-fold too high. Crandall *et al.*[25] calibrated population expansion signals of several marine taxa to inferred post-LGM habitat expansion and obtained mtDNA rates ca. 7-fold higher than conventionally assumed, and Grant *et al.*[18] specifically criticized conventional dating of “native” Pacific herring population history on similar grounds.

Notably, attempts to date trans-Arctic invasion events by external mtDNA rates and coalescence methods (including IM and BSL) now consistently result in estimates falling to the Middle Weichselian, i.e. to the most improbable time frame, midway between the current (< 12 kya) and previous (120 kya) interglacials, when trans-Arctic connections were closed: most estimates in each of *Clupea, Mytilus* and *Macoma* ranged 20–70 kya. A notion of e.g. 7-fold underestimate of recent rate would indeed bring all these conveniently close to the early-to-middle Holocene warm period 5–10 kya. While this is reassuring, the modified estimates evidently cannot have any accuracy, which further stresses the qualitative nature of the inference from the single-locus coalescence analyses. Yet, by a simple reasoning based on upper limits of *u* and the inferred (minimum) number of mutations from star-phylogeny components of the haplotype diagrams, we obtain minimum ages that reliably bracket out the most recent time frame affected by human activities.

The mechanisms underlying the time-dependent rate are not well understood (see [18]). The phenomenon entails the failure of the applied substitution models to linearize the evolutionary time scale. While the time-dependency has been recorded from studies of both coding and non-coding mtDNA, the substitution models could be expected to deal better with the coding gene evolution (mainly of silent sites) than that of the *CR*, often structurally constrained and involving mutation hotspots. To explore this expectation, we compared the biases resulting from the analysis of the coding vs. *CR* segments in the same mtDNA molecule genealogy in our herring data: the critique of herring time estimates by Grant *et al.*[18] primarily concerned *CR* estimates.

While the demographic signals in the mismatch and BSL plots from the two markers appear broadly similar (Figure 5), the quantitative estimates (Additional file 3: Table S1) and a *cyt-b* vs. *CR* plot of selected diversity and divergence estimates (Additional file 2) indeed do show some consistent differences, which are not always in the expected direction. From a “calibration” fixed at the inter-species TMRCA, the modeled rate for *CR* is 3.6 times faster than of *cyt-b* (16.9% vs. 4.7% divergence; the substitution models adopted for the two regions were indeed quite similar). Applying these rates at shorter distances however yields consistently younger ages from *CR* than from *cyt-b* (Figure 5; Additional file 2). The models thus appear to do better in linearizing the *CR* than the *cyt-b* divergence scales. Actually, from the mismatch and BSL approaches, the “conventional” *CR* estimates for the European invasion/expansion would point very close to the post-glacial warm period (5–13 kya), whereas *cyt-b* estimates mostly fall to pre-LGM times > 20 kya, whereby it appears that there would be little if any bias in the *CR* data at these time frames. Grant *et al.*[18] in turn still suggested a 3-fold acceleration in their Pacific herring *CR* data, reflecting the intra-basin expansion signal in the Pacific. In more concrete terms, it seems that the inferred post-glacial bursts of new haplotypes that manifest the accelerated rate are less extensive in North European *CR* than *cyt-b* data, but such inter-locus difference is not seen in the NWP populations even in our data (Figure 3). This would suggest even population-wise or regional differences in the (ir)regularity of the rate.

## Conclusions

Patterns of mitochondrial diversity in the Pacific and Atlantic herrings *C. pallasii* and *C. harengus* reflect a vicarious history in the two ocean basins, with similarly large long-term effective sizes in both species but contrasting patterns of intra-basin subdivision. The long-term inter-oceanic vicariance of the two species is broken by a presence of outpost Pacific herring populations in NE Europe, which are inferred to represent early post-glacial colonizers derived entirely from the Asian-Beringian *C. pallasii* clade. The history of this secondary invasion has involved bottlenecks and has been followed by accumulation of new mtDNA variation. A strong regional substructure has evolved since that time in Europe, in stark contrast to the broad-scale uniformity maintained by herrings in their native basins. This structure only partly matches the previous biological concepts based on seasonal breeding stocks or geographical subspecies designations. The observed trans-Arctic herring phylogeography is notably similar to those of two amphi-boreal mollusk taxa, *Macoma* and *Mytilus,* suggesting similar histories of inter-oceanic connections, and plausibly similar responses to and genetic consequences from future changes in Arctic hydrography. We also considered the suggested time dependency of molecular rates, critical for interpreting timing of relatively recent biogeographical events, by directly comparing estimates from coding and non-coding mitochondrial regions of presumably different mutation dynamics.

### Availability of supporting data

The data sets supporting the results of this article are available in the GenBank (accession numbers KC599333-KC599541 (*cyt-b*) and KC594362-KC594546 (*CR*)) and Dryad repository (doi: http://dx.doi.org/10.5061/dryad.q31f8). GenBank: sequence data. Dryad: list of individual samples with sample locations and Genbank accession numbers for both gene regions, Arlequin input files with population grouping, IM input files, pairwise interpopulation *Φ*_ST_ distance matrix for the MDS ordination.

## Competing interests

The authors declare that they have no competing interests.

## Authors' contributions

RV designed the study, DLL, PS, HML and RV organized sampling. HML performed molecular labwork and conducted data analyses. HML and RV prepared the manuscript, with expert contributions from DLL and PS. All authors read and approved the final manuscript.

## Supplementary Material

Additional file 1 Details of molecular and statistical analyses. Click here for file

Additional file 2: Figure S1 Comparing the performance of GTR+I+Γ model distance correction in the coding and non-coding gene segments. Click here for file

Additional file 3 **Table S1.** Age estimates for demographic events from mismatch distributions and from Bayesian skyline plot analysis (summarizing inferences from the analyses presented in Figure 5.). **Table S2**. IM-model analysis results (a more detailed version of the results presented in Table 4 of the paper). Click here for file

Additional file 4: Figure S2 Neighbor-joining tree of mtDNA haplotypes from *CR* data (GTR+I+Г model distances; comparable figures for *cyt-b* and concatenated data are presented in the paper itself). Click here for file
