# Supplementary material for: Phylogeography of amphi-boreal fish: tracing the history of the Pacific herring Clupea pallasii in North-East European seas
Source: BMC Evol Biol. 2013 Mar 19;13:67. doi: 10.1186/1471-2148-13-67 (PMC3637224; doi:10.1186/1471-2148-13-67)
Supplement: Additional file 1 — Details of molecular and statistical analyses. [file 1471-2148-13-67-S1.pdf]

Laakkonen HM, Lajus DL, Strelkov P, Väinölä R (2013) BMC Evol Biol

Additional file 1: Details of molecular and statistical analyses

1. Amplification and sequencing of mitochondrial DNA

Primers used for P*CR* amplification and sequencing of two mtDNA regions were:

A. Universal fish cytochrome-b PCR primers [1]:
L15361 AAACTAATGACTTGAAAAACCACCGTTG
H16553 TTAACCTCCGATCTCCGGATTACAAGAC
B. Internal cytochrome-b sequencing primers for *Clupea* spp. (original):
Cytb-cluiL GTTCCSTATGTAGGTAACGCAC
Cytb-cluiH GGAGTTAAGTCCYGCCGGGTTG
C. Control region primers for *Clupea* spp. (original):
L19c TACCCCTAACTCCCAAAGCTA
ClupeaCRr TGACCCTTATCATTCATGATATGC

Each P*CR* reaction mixture (volume 20 μl) contained 50–200 ng total genomic DNA as template, 5 pmol each primer, 200 μM dNTPs, 1×PCR buffer, 2 mM $MgCl_2$ and 1 unit of Taq DNA polymerase (reaction chemicals manufactured by Fermentas). Amplification was performed with Eppendorf Mastercycler EP Gradient S 96 for one denaturation step at 95°C for 2 min, followed by 35 cycles at 95°C for 50 s, 55°C for 50 s, and 70°C for 1 min 20 s; and a final 7 min extension step at 72°C. PCR products were purified with ExoSAP-IT® (Fermentas) protocol. BigDye™ 1.1 (Applied Biosystems) was used for sequencing, and products were purified with Sephadex™ columns (GE Healthcare Life Sciences). Sequences were resolved on MegaBACE 1000 96-capillary systems.

2. Substitution models

The best-fit substitution model for each or the *cyt-b*, *CR* and concatenated datasets was selected by the procedure in Modeltest 3.7 [2] applied to the full two-species data. The GTR+I+Γ model (General Time Reversible model, with a category of invariable sites and gamma distribution of rate variation) was chosen for each set. For *cyt-b*, the estimate of the proportion of invariant sites was p-inv = 0.52 and gamma distribution shape parameter $\alpha = 0.63$; for *CR*, p-inv = 0.59 and $\alpha = 0.70$; for the concatenated data p-inv = 0.64 and $\alpha = 0.62$.

3. Coalescence-based analyses of population history

IM: Estimating demographic parameters in a two-population divergence model
The MCMC simulation program IM [3] was applied to *cyt-b* data of population pairs representing the trans-Arctic lineage. The pooled NW Pacific samples were contrasted separately to each of the three genetically defined European population groups (White Sea, Mezen-Pechora and Balsfjord). For the two-population system, the program looks for HPD (highest probability density) estimates for a number of demographic parameters in a Bayesian setting: the ancestral population mutation parameter θ_A ($=N_A u$, a measure of diversity and population size), the current population parameters $\theta 1$ and $\theta 2$, population splitting time t, the splitting ratio, and post-split immigration rates $m1$ and $m2$.

Uniform priors for the relevant parameters were used, and the upper limits of prior distributions were decided by screening in preliminary runs as suggested by Won and Hey [4]. The HKY substitution model and a burn-in period of 2 x 10^6 steps was used in the simulations (run parameter settings: -q1 1000 -q2 1000 -qA 100 -m1 10 -m2 2 -t 10 -u -b 1 000 000 -fg -g1 0.95 -g2 0.8 -n 40 -k 40 -j9 -j1). There was not enough information in the data to yield estimates of population size related parameters $\theta 1$ and $\theta 2$ which fluctuated till infinity in the preliminary runs. Despite varying prior upper limits set for these parameters in the preliminary runs, and the fluctuating results, the HPD values of other parameters remained relatively stable (deviations well within the 90% HPD interval; see Table 4), and thus were considered reliable or at least indicative. In the final runs, prior upper limits for the $\theta 1$ and $\theta 2$ parameters were set based on the effective population sizes inferred from Bayesian skyline analysis (Figure 5). A geometric heating scheme was used with 40 chains and 40 swap attempts per step, in order to reach true mixing in the model. Three separate runs with over 2 000 000 genealogies were made to assess consistency across multiple runs.

BEAST: Bayesian skyline plot analysis of population size history

The Bayesian skyline plot analysis of population size history, using BEAST v1.5.3 software [5] was applied separately to the *cyt-b* and *CR* sequences, for various subsets of data representing genetically homogeneous population groups. To test for convergence, 10^8 iterations were performed with a burn-in of 10^7 under the GTR+I+Γ model (or 5 x 10^9 iterations with 5 x 10^8 burn-in when needed; for White Sea and Mezen–Chesha), a strict molecular clock and a stepwise skyline model with 20 piecewise intervals in the genealogies for *C. harengus* and for the NE Pacific and NW Pacific *C. pallasii*, and with 5 intervals for the European *C. pallasii* subgroups. Genealogies and model parameters were sampled every 10 000 iterations and operators were optimized automatically. Effective sample size (ESS) for each parameter exceeded 200. The trajectories were plotted by Tracer v1.5 (included in the BEAST package). The time axis (in terms of estimated divergence) was related to calendar years with using the long-term rates below. The population size axis was scaled assuming a 4-yr generation time [6].

Mismatch distributions

Expansions within population groups judged to represent historically coherent entities were illustrated in mismatch distributions separately for *cyt-b* and *CR* data using Arlequin ver. 3.5 [7]. As the mismatch distributions were often not simply unimodal, the timing of the possible expansion events were judged directly from the prominent peaks in the distributions. An approximate model correction for the peak age was made, by applying the relationship (ratio) of the model estimate vs. observed distances of corresponding age in the actual individual or average distances in the data (e.g. those plotted in Additional file 3). The most recent expansion represented by the zero modes (in the trans-Arctic populations) was however calculated directly as an average from the first two bars in the distribution (0 and 1 differences).

4. Long-term molecular rates

Conventionally, molecular datings have been based on rates derived from fossil or biogeographical calibration points of "deep", pre-Pleistocene age (>2 Myr). Whereas it is now clear that such rates are not valid on short time scales (see Discussion: Non-linear rate and amending of time estimates), we use a tentative 1.5 % My^{-1} (0.75% per lineage) rate as an operational scale for the initial discussion. There are no direct calibration points even for the deep rate in *Clupea*, but this rate is used on two general arguments. First, on general biogeography, the Pacific and Atlantic herrings represents a

general pattern of inter-oceanic vicariance observed in numerous taxa and thought to be a result of the Great Trans-Arctic Interchange that followed the Pliocene opening of the Bering Strait [8]. A date of 3.5 Mya, related to the evidence of the first appearance Pacific mollusks in the Atlantic has most often been used as a calibration date for the various vicarious taxa (e.g. [9-12]), while the time favourable for exchange could plausibly range some 5.4 to 2.5 Mya, i.e. from opening of the strait to start of Pleistocene glaciations [8,13]. Second, on comparative fish data: Kontula *et al.* [1] cited mitochondrial coding-gene rates estimated from a number of taxa based on fossil or biogeographical evidence, ranging 0.5–1.2% divergence My^{-1}, and external rates 1.0–2.7% My^{-1} have been applied in a number of recent fish studies [1,14-17]. The 1.5 % My^{-1} *cyt-b* rate fits this range and also the popular trans-Beringian biogeographical age (4.7 % lineage divergence *C. pallasii* and *C. harengus* lineage divergence corresponding to 3.1 My, or roughly 3.6 % net divergence to c. 2.4 My population split). The estimated interspecific *CR* divergence was 3.6 times that in *cyt-b*, and if the substitution models actually would properly linearize the divergence scales, the *CR* rate would be 3.6 fold, i.e. 5.4% My^{-1}.

References

1. Kontula T, Kirilchik SV, Väinölä R: **Endemic diversification of the monophyletic cottoid fish species flock in Lake Baikal explored with mtDNA sequencing.** *Mol Phylogenet Evol* 2003, **27**(1):143-155.
2. Posada D, Crandall KA: **MODELTEST: testing the model of DNA substitution.** *Bioinformatics* 1998, **14**(9):817-818.
3. Hey J, Nielsen R: **Multilocus methods for estimating population sizes, migration rates and divergence time, with applications to the divergence of *Drosophila pseudoobscura* and *D. persimilis*.** *Genetics* 2004, **167**(2):747-760.
4. Won YJ, Hey J: **Divergence population genetics of chimpanzees.** *Mol Biol Evol* 2005, **22**(2):297-307.
5. Drummond AJ, Suchard MA, Xie D, Rambaut A: **Bayesian phylogenetics with BEAUti and the BEAST 1.7.** *Mol Biol Evol* 2012, **29**: 1969–1973.
6. Dmitriev NA: **Biology and Fishery of Herring in the White Sea.** Moscow: Pishchepromizdat; 1946.
7. Excoffier L, Lischer HEL: **Arlequin suite ver 3.5: a new series of programs to perform population genetics analyses under Linux and Windows**. *Mol Ecol Resour* 2010, **10**(3):564-567.
8. Vermeij GJ: **Anatomy of an invasion - the trans-Arctic interchange.** *Paleobiology* 1991, **17**(3):281-307.
9. Bowen BW, Bass AL, Rocha LA, Grant WS, Robertson DR: **Phylogeography of the trumpetfishes (*Aulostomus*): Ring species complex on a global scale.** *Evolution* 2001, **55**(5):1029-1039.
10. Nikula R, Strelkov P, Vainola R: **Diversity and trans-Arctic invasion history of mitochondrial lineages in the North Atlantic *Macoma balthica* complex (Bivalvia: Tellinidae).** *Evolution* 2007, **61**(4):928-941.
11. Rawson PD, Harper FM: **Colonization of the northwest Atlantic by the blue mussel, *Mytilus trossulus* postdates the last glacial maximum.** *Mar Biol* 2009, **156**(9):1857-1868.
12. Liu J-X, Tatarenkov A, Beacham TD, Gorbachev V, Wildes S, Avise JC: **Effects of Pleistocene climatic fluctuations on the phylogeographic and demographic histories of Pacific herring (*Clupea pallasii*).** *Mol Ecol* 2011, **20**(18):3879-3893.
13. Marincovich L: **Central American paleogeography controlled Pliocene Arctic Ocean molluscan migrations.** *Geology* 2000, **28**(6):551-554.
14. Bigg GR, Cunningham CW, Ottersen G, Pogson GH, Wadley MR, Williamson P: **Ice-age survival of Atlantic cod: agreement between palaeoecology models and genetics.** *Proc R Soc B* 2008, **275**(1631):163-173.
15. Carr SM, Marshall HD: **Phylogeographic analysis of complete mtDNA genomes from Walleye Pollock (*Gadus chalcogrammus* Pallas, 1811) shows an ancient origin of genetic biodiversity.** *Mitochondr DNA* 2008, **19**(6):490-496.
16. Grant WS LM, Gao TX, Yanagimoto T: **Limits of Bayesian skyline plot analysis of mtDNA sequences to infer historical demographies in Pacific herring (and other species).** *Mol Phylogenet Evol* 2012, **65**(1):203-212.
17. Lessios HA: **The great American schism: divergence of marine organisms after the rise of the Central American Isthmus.** *Annu Rev Ecol Evol Syst* 2008, **39**:63-91.
