# Supplementary material for: Phylogeography of amphi-boreal fish: tracing the history of the Pacific herring Clupea pallasii in North-East European seas
Source: BMC Evol Biol. 2013 Mar 19;13:67. doi: 10.1186/1471-2148-13-67 (PMC3637224; doi:10.1186/1471-2148-13-67)
Supplement: Additional file 2: Figure S1 — Comparing the performance of GTR+I+Γ model distance correction in the coding and non-coding gene segments. [file 1471-2148-13-67-S2.pdf]

Laakkonen HM, Lajus DL, Strelkov P, Väinölä R (2013) *BMC Evol Biol*

Additional file 3: Figure S2: Comparing the performance of GTR+I+Γ model distance correction in the coding and non-coding gene segments

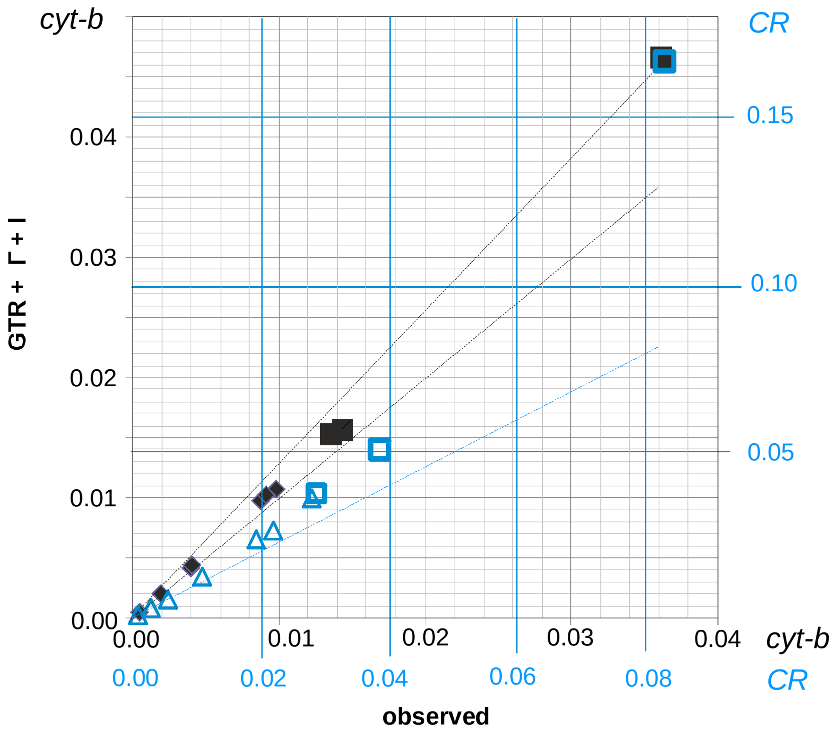

Figure S2: Comparing the performance of GTR+Γ+I model distance correction in the coding and non-coding gene segments
The GTR+Γ+I model distance correction in the coding (*cyt-b*, filled symbols) and non-coding (*CR*, open blue symbols) gene segments when "deep calibration" by interspecies reference is used. For each gene, observed vs. corrected distances are plotted. The corrected GTR+I+Γ values are from Table 2, the corresponding observed values are close to the π diversity values: diamonds and triangles are the group-wise averages for the geographical subsets, the squares are inter- and intraspecies basal distances. The markers are plotted on different scales (black for *cyt-b*, blue for *CR*) so that the interspecies distance estimates coincide (upper right corner). The figure illustrates that if the interspecies distance is used for setting a time scale, and if a time-dependence of the rate is expected to cause age overestimation of recent events, such a bias would be considerably smaller for CR than for *cyt-b*, even if *CR* generally evolves faster and is thought to show more irregular patterns.
