# Supplementary material for: Phylogeography of amphi-boreal fish: tracing the history of the Pacific herring Clupea pallasii in North-East European seas
Source: BMC Evol Biol. 2013 Mar 19;13:67. doi: 10.1186/1471-2148-13-67 (PMC3637224; doi:10.1186/1471-2148-13-67)
Supplement: Additional file 3 — Table S1. Age estimates for demographic events from mismatch distributions and from Bayesian skyline plot analysis (summarizing inferences from the analyses presented in Figure 5.). Table S2. IM-model analysis results (a more detailed version of the results presented in Table 4 of the paper). [file 1471-2148-13-67-S3.pdf]

Additional file 4: Tables S1 and S2

Table S1: Age estimates for demographic events from mismatch distributions and from Bayesian skyline plot analysis (summarizing inferences from the analyses presented in Figure 5.). Mean pairwise differences and inferred calendar years from Figure 5, assuming the operational long-term per-site substitution rates of u = 0.75% My^{-1} (cyt-b) and 2.7% My^{-1} (CR). The mismatch signals were taken directly from the prominent peaks in the distributions. The most recent expansion represented by the zero modes was however calculated directly as an average from the first two bars (0 and 1 differences). The BSL signals were determined visually from the graphs, as the starting point of a prominent rise in the trajectory. The deepest BSL signal corresponds to the basal coalescence (median estimate of the root). The difference estimates (%) for mismatch plots are observed values, those for BSL from the model-corrected scale. The “age” estimates for both are corrected (see Additional file 1).

	Mismatch peaks							BSL expansion signals						
Genetic group	Cytochrome b			Control region				Cytochrome b			Control region			
Balsfjord														
Difference (%)	0.019	-	-	0.044	-	-	-	0.02	-	-				
Age (kyr)	26	-	-	16	-	-	-	27	-	-				
White Sea														
Difference (%)	0.013	0.27	-	0.014	0.41	-	-	0.03	0.2	-	0.04	0.37	-	-
Age (kyr)	18	370	-	5	160	-	-	33	270	-	13	140	-	-
Mezen–Chesha														
Difference (%)	0.015	0.27	-	0.015	0.41	-	-	0.03	0.4	-	0.04	0.45	-	-
Age (kyr)	20	380	-	6	160	-	-	33	530	-	13	170	-	-
NW Pacific														
Difference (%)	(0.018)	0.18	-	(0.030)	0.41	0.82	-	0.04	0.27	-	0.10	1.00	1.30	-
Age (kyr)	(25)	250	-	(11)	160	350	-	53	360	-	37	370	480	-
NE Pacific														
Difference (%)	-	0.27	0.67	-	-	0.72	1.34	0.05	0.34	0.69	0.10	-	1.00	2.10
Age (kyr)	-	400	990	-	-	350	610	67	450	920	37	-	370	770
C. harengus														
Difference (%)	-	0.31	0.57	-	-	0.93	1.4–1.7	-	0.37	0.74	-	-	1.80	3.90
Age (kyr)		450	830	-	-	440	700–800	-	490	990	-	-	670	1430

Table S2: IM-model analysis results (a more detailed version of the results presented in Table 4 of the paper). IM-model based analysis of Pacific-Atlantic relationships from coalescence simulations on the trans-Arctic clade cyt-b data. θ = population mutation parameter describing the population size and diversity in the NWP, European and Ancestral populations; s = population splitting parameter, m = gene flow ($m1$ = migration to NWP, $m2$ = migration to Europe) and t time since divergence in absolute mutation units. Effective population sizes N_e and time of divergence in calendar years are based on the assumed per gene mutation rate 8.48 % My^{-1}; N_e are also scaled assuming a 4-yr generation time (Dmitriev NA: Biology and Fishery of Herring in the White Sea. Moscow: Pishchepromizdat; 1946).

Comparison	θ_{NWP}	θ_{Eur}	θ_A	1 - s	$m1$	$m2$	t	N_{eNWP} x 10^6	N_{eEur} x 10^6	N_{eA}	t (ky)
NW Pacific vs:											
-White Sea group	986	954	74	0.80 %	2.98	0	0.44	7.3	7	540 000	52
Lower 90% HPD	484	535	47	2.90 %	0.94	0	0.30	3.6	3.9	350 000	36
Upper 90% HPD	[1000]	[1000]	109	0.00 %	5.47	0.4	0.63	7.4	7.4	800 000	75
-White Sea group[b]	938	100	54	1.80 %	1.33	0.14	0.59	6.9	0.74	400 000	70
Lower 90% HPD	274	84	27	8.20 %	0.86	0	0.57	2.0	0.62	200 000	67
Upper 90% HPD	[1000]	[100]	96	0.60 %	2.75	0.4	1.49	7.4	0.74	710 000	175
-Mezen–Chesha group[a]	987	18	83	1.10 %	4.99	0	0.43	4.4	0.13	610 000	51
Lower 90% HPD	476	8	46	6.90 %	1.45	0	0.25	3.5	0.06	340 000	29
Upper 90% HPD	[1000]	[1000]	129	0.00 %	8.93	0.71	1.50	7.4	7.4	950 000	177
-Balsfjord[a]	760	6	41	0.05 %	0.65	0	*	5.6	0.04	300 000	-
Lower 90% HPD	531	2	9	51.30 %	0.1	0	*	3.9	0.01	60 000	-
Upper 90% HPD	[1000]	92	95	0.00 %	1.19	0.7	*	7.4	0.67	700 000	-

[a] Upper limits for prior distribution of θ set to 1000.

[b] Upper limits for prior distribution of θ were set to θ_{NWP}=1000, θ_{Eur}=100 and θ=100.

*The analysis did not converge.
