# Supplementary material for: Phylogeography of amphi-boreal fish: tracing the history of the Pacific herring Clupea pallasii in North-East European seas
Source: BMC Evol Biol. 2013 Mar 19;13:67. doi: 10.1186/1471-2148-13-67 (PMC3637224; doi:10.1186/1471-2148-13-67)
Supplement: Additional file 4: Figure S2 — Neighbor-joining tree of mtDNA haplotypes from CR data (GTR+I+Г model distances; comparable figures for cyt-b and concatenated data are presented in the paper itself). [file 1471-2148-13-67-S4.pdf]

Laakkonen HM, Lajus DL, Strelkov P, Väinölä R (2013) *BMC Evol Biol*

Additional file 2: Figure S1: Neighbor-joining tree of mtDNA haplotypes from CR data.

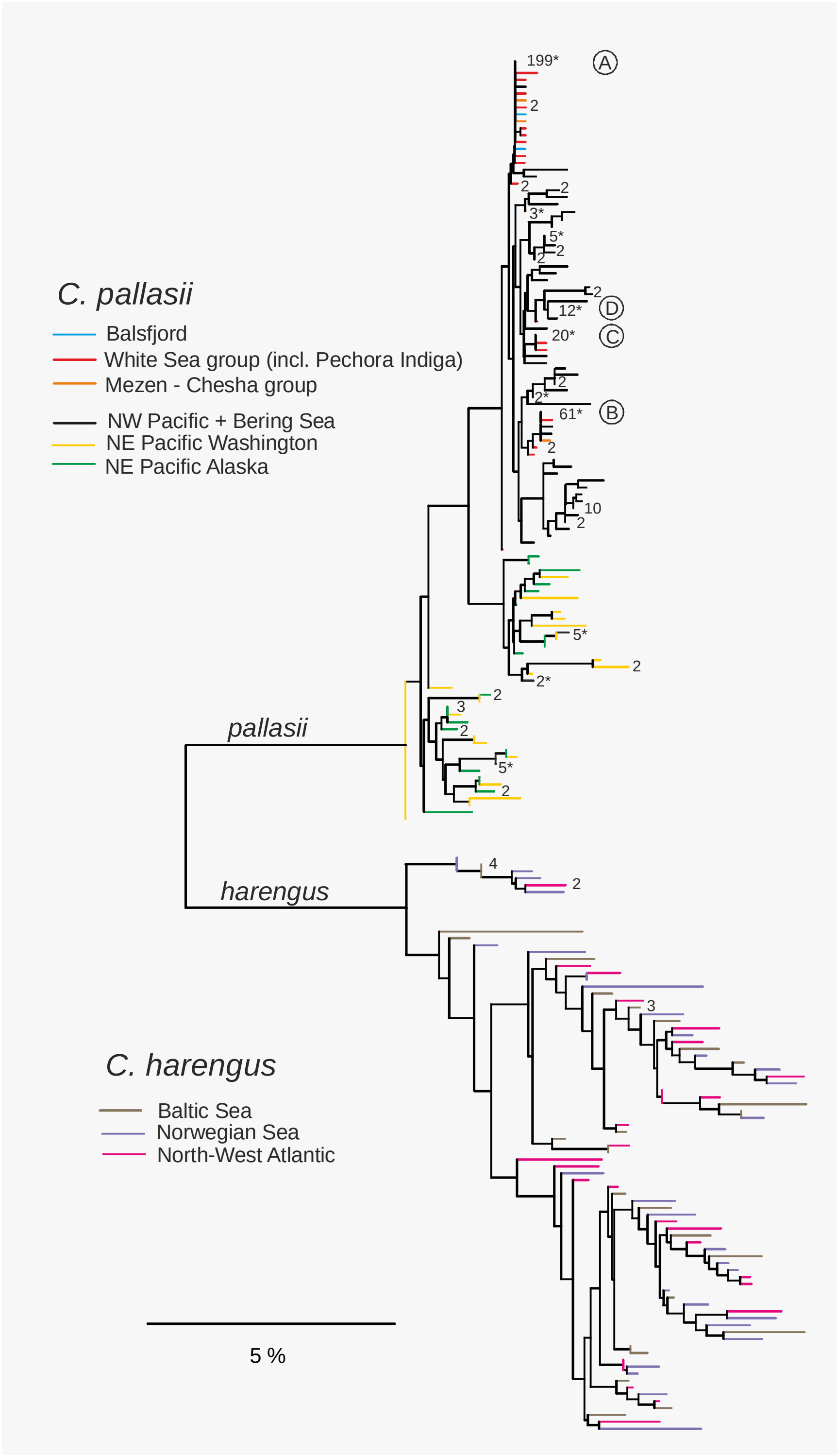

Figure S1: Neighbor-joining tree of mtDNA haplotypes from CR data. The tree is based on GTR+I+Γ model distances, and is comparable to the figures for *cyt-b* and concatenated data presented in Figure 2 of the paper itself. The numbers are observed frequencies of haplotypes found in multiple individuals; haplotypes shared between localities are indicated with asterisks. Color codes refer to population groups as in Figure 1. The core haplotypes A-D from Figure 3 are indicated.
